# Use of a Semiautomatic Text Message System to Improve Satisfaction With Wait Time in the Adult Emergency Department: Cross-sectional Survey Study

**DOI:** 10.2196/34488

**Published:** 2022-09-06

**Authors:** Frederic Ehrler, Jessica Rochat, Johan N Siebert, Idris Guessous, Christian Lovis, Hervé Spechbach

**Affiliations:** 1 Division of Medical Information Sciences University Hospitals of Geneva Geneva Switzerland; 2 Faculty of Medicine University of Geneva Geneva Switzerland; 3 Department of Pediatric Emergency Medicine Geneva Children’s Hospital Geneva University Hospitals Geneva Switzerland; 4 Division of Primary Care Geneva University Hospitals Geneva Switzerland; 5 Department of Health and Community Medicine Faculty of Medicine University of Geneva Geneva Switzerland; 6 Ambulatory Emergency Care Unit Department of Primary Care Medicine Geneva University Hospitals Geneva Switzerland

**Keywords:** emergency, patient satisfaction, service-oriented health care, quality of care, health service, emergency department

## Abstract

**Background:**

Many factors influence patient satisfaction during an emergency department (ED) visit, but the perception of wait time plays a central role. A long wait time in the waiting room increases the risk of hospital-acquired infection, as well as the risk of a patient leaving before being seen by a physician, particularly those with a lower level of urgency who may have to wait for a longer time.

**Objective:**

We aimed to improve the perception of wait time through the implementation of a semiautomatic SMS text message system that allows patients to wait outside the hospital and facilitates the recall of patients closer to the scheduled time of meeting with the physician.

**Methods:**

We performed a cross-sectional survey to evaluate the system using a tailored questionnaire to assess the patient perspective and the Unified Theory of Acceptance and Use of Technology questionnaire for the caregiver perspective. We also monitored the frequency of system use with logs.

**Results:**

A total of 110 usable responses were collected (100 patients and 10 caregivers). Findings revealed that 97 of 100 (97%) patients were satisfied, with most patients waiting outside the ED but inside the hospital. The caregiver evaluation showed that it was very easy to use, but the adoption of the system was more problematic because of the perceived additional workload associated with its use.

**Conclusions:**

Although not suitable for all patients, our system allows those who have a low-severity condition to wait outside the waiting room and to be recalled according to the dedicated time defined in the Swiss Emergency Triage Scale. It not only has the potential to reduce the risk of hospital-acquired infection but also can enhance the patient experience; additionally, it was perceived as a real improvement. Further automation of the system needs to be explored to reduce caregiver workload and increase its use.

## Introduction

### Background

Patients triaged with low priority in the emergency department (ED) are likely to have a long wait before being seen by a physician, as those with life-threatening and serious conditions are prioritized over patients that are less acute [1]. A side effect of long wait times is the risk that patients leave the ED without being seen by a physician, with this risk increasing significantly after a 1-hour wait [2]. It has also been shown that long wait times can result in staff interruptions by frustrated patients and lead to violent behavior [3,4]. Additionally, it has been reported that a long wait time increases the risk of contracting hospital-acquired infections [5]. As an example, Beggs et al [6] showed that the number of new cases of airborne infections increased substantially with time spent in the waiting room and the number of people waiting. However, reducing overcrowding in the ED waiting room is not a simple task [7]. The space available is often limited and the nature of the ED does not allow for a control on its occupation, which varies significantly over the course of a single day [8,9].

Several attempts have been performed to improve the wait time experience in the ED, either by minimizing the duration between triage and patient care or by acting on the actual perception of wait time [7,10]. Although organizational measures can improve ED efficiency, such as fast track [11], improved triage [12], and better team communication, they will never prevent overcrowding situations, as ED staff cannot be adjusted as quickly as the influx evolves. By contrast, improving the patient experience during the wait has been favored through interventions such as providing information to the patient about the expected wait duration [13], comfort improvement in the waiting room [14], or giving a pager to patients, which allowed them to wait in a place other than the waiting room [15]. These interventions were shown to have a positive impact and are promising strategies to be further explored.

To reduce ED congestion and improve the perception of wait time, we developed a semiautomated message (SMS text message) system that allows patients to wait outside the emergency waiting room and to be recalled closer to the actual time of the medical consultation. In this study, we explore the perceptions of this system by patients and caregivers.

### A Semiautomatic SMS Text Message System

**Figure 1:**
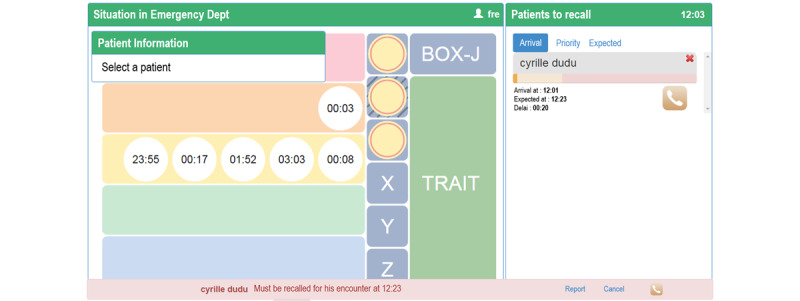
Main screen of the SMS text message recall system. The left-hand side represents the waiting queue in the emergency department waiting room, with each line representing the emergency level and each circle a patient currently waiting. The vertical middle row represents the emergency rooms and their occupancy, with each patient also represented by a circle. The right-hand side is the SMS text message recall system. The patients enrolled are presented with information on their arrival time and expected meeting time with the physician.

The system was initially developed at the pediatric department of Geneva University Hospitals (Geneva, Switzerland) [16], adapted later for the adult setting and deployed in September 2017, and finally introduced in the gynecology and obstetrics setting in 2019. It aims at improving patient flow in EDs by providing caregivers with a system to monitor the flow and ED occupancy. The system allows triaged patients with a low-severity grade to wait outside the ED and to be called back by a recall SMS text message system shortly before they are to be seen by a physician. A screen available to nurses provides real-time occupancy of the emergency rooms and wait times by triage level (Figure 1).

Once triaged, each patient can be registered in the SMS text message system by a nurse. A screen displays the patient’s key administrative information, allowing the administrative clerk to verify the validity of the patient’s telephone number. The nurse estimates the length of the wait and validates the patient’s registration in the system. The patient then receives a confirmation message and can leave the ED while remaining virtually in the queue. Whether they are physically present or not, all patients are moved forward normally in the queue and recalled based on their arrival time and emergency level. All registered patients waiting outside the ED are visible on a screen with a time bar individually associated with them and showing the expected time to being seen by a physician. The time bar progressively changes color based on the elapsed time and actions that need to be taken by the caregiver responsible for calling the patient back. A green bar indicates that no recall is needed yet since the meeting with the physician is still distant. The bar turns orange 20 minutes before the patient’s scheduled return, suggesting that the triage nurse call the patient back, without being mandatory. If the scheduled return time has passed, the bar turns red.

Dispatch of the first recall SMS text message is left to the discretion of the triage nurse to determine the most opportune time to return for the visit. If the patient does not arrive within 20 minutes of the first SMS text message, the system is automated to send reminder messages every 20 minutes (total of four SMS text messages). At any time, the nurse has the possibility to inform the patient about the evolution of the situation at the ED by sending a predefined message to the patient. Depending on the situation, the message sent will inform the patient that the visit is postponed due to a strong influx of patients or that an early return is possible due to an improved situation. If the patients do not arrive after three reminder SMS text messages, a final fourth SMS text message is sent to inform them that the position in the queue is no longer guaranteed, but the visit is still possible.

## Methods

### Study Design

This study is a cross-sectional descriptive investigation using a mixed methods methodology, including an assessment of the patients’ experience during their wait in the ED through a tailor-made questionnaire, analysis of the system log to understand the use trend, and an assessment of nurses’ acceptance of the SMS text message recall system. The survey was conducted between March 13 and April 28, 2017, at the 24-hour ED outpatient unit at Geneva University Hospitals, the largest public hospital in Switzerland with 70,000 patient admissions each year. Utilization logs were collected from October 2017 to August 2019.

### ED Setting: Emergency Outpatient Unit

Medical and traumatic pathologies are treated in 12 consultation rooms. Patients wait in a semienclosed waiting room with seating, a television, water, and newspapers. The staff (clinicians and nurses) are the same for the entire unit. The median length of stay is 3.5 hours, with a median waiting time of 1.5 hours.

When patients arrive at the main ED entrance, they are first seen by a triage nurse who decides whether the patient is a candidate for the outpatient unit, based on the Swiss Emergency Triage Scale [17]. Level 1 is a life-/limb-threatening situation where the patient must be seen immediately by a physician. Level 2 must be seen within 20 minutes, level 3 within 120 minutes, and level 4 is considered nonurgent. A total of 80% of patients who come to the ED are classified as level 3 and 10% as level 4. After triage, the patient goes through an administrative registration process and is then directed to one of the subunits by following colored lines on the floor. These lead to a nurse’s desk where a nurse escorts the patient to the waiting room. Whenever possible, the nurses inform patients of the estimated waiting time. As soon as a consultation room and physician are available, the patient is taken to the room by the nurse. After the medical visit, the patient can either go home or may have to undergo an additional examination and return to the waiting room. A small percentage of patients (8%) are hospitalized and 5% leave the unit without being seen by a physician [18]. The probability of leaving the ED prematurely is linked to flow concern as demonstrated in previous studies [19].

### Study Participants

Patients presenting to the ED outpatient unit with a triage level of 3 and 4 (according to the 4-level Swiss Triage Scale) were invited to participate in the questionnaire part of the study if they were 16 years of age or older and spoke French. We used a convenience sampling method and arbitrarily defined the sample size as 100 participants. Exclusion criteria were patients not capable of discernment (eg, unconscious, intoxicated, extreme trauma, or cognitive impairment), unable to read/understand French, vision problems, severe pain or overly aggressive, and those who had already completed the questionnaire.

### Measurement Instruments

#### Patient Satisfaction Questionnaire

A 12-item questionnaire was designed to assess the patient experience among those who had used the SMS text message system. This questionnaire was of our own design. It contained an item aiming to determine the minimum expected wait time before patients find the system useful. It also explored where the patient waited until being taken care of, whether the advertised waiting time matched the actual waiting time, and whether the content of the SMS text message was clear. Users were asked if they felt stressed during the wait, if they had enough time to come back to the emergency room, and if they were satisfied with the system overall. In addition, the actual wait time for each patient who completed the questionnaire was extracted from the hospital clinical information system.

#### Caregiver Acceptance Questionnaire

The 21-item Unified Theory of Acceptance and Use of Technology (UTAUT) questionnaire is a unified technology acceptance model formulated by Venkatesh et al [20] as a conceptual framework to understand users’ intended use and acceptance of new information technologies, which can be determined by 5 constructs: (1) performance efficiency (4 questions), (2) effort expectancy (4 questions), (3) social influence (4 questions), (4) facilitating conditions (4 questions), and (5) behavioral intention to use the system in the future (3 questions). Each question is scored on a 7-point Likert scale. The questionnaire was distributed anonymously to all nurses working with the system.

#### System Use Logs

System use was assessed by analysis of the system use logs. A log, including a time stamp, was generated each time a caregiver entered a patient into the SMS text message system, as well as each time a SMS text message was sent.

### Procedure and Ethical Considerations

The Geneva Institutional Ethics Committee approved the study protocol (Réq-2016-00555). Patient participation in the study was voluntary, and oral consent was obtained prior to the intervention. After verification of the inclusion criteria, the nurse asked the patients if they agreed to use the SMS text message recall system. Information about the study and confidentiality were given verbally. If accepted, the patients were allowed to wait wherever they wanted (ie, in or outside the ED). We did not verify where the patient waited as it would have been difficult to trace. We arbitrarily decided to set the number of questionnaires to be completed at 100.

Once back in the ED, the patient was immediately brought to a consultation room. The patient was given the study questionnaire by a nurse while waiting for the physician. The nurse remained available for any questions and to assist the patient in completing the questionnaire if necessary. Instructions were given to the medical staff to see the patients immediately after completion of the questionnaire. Once completed, questionnaires were collected by nurses and placed in a dedicated box in a secure room. Questionnaires were collected each morning by a scientific collaborator, and the responses were entered into an Excel (Microsoft Corporation) file. To link the questionnaire data to data extracted from the hospital clinical information system, we used a mapping file linking the questionnaire ID to the patient ID. Once all data were included in the Excel file, only the questionnaire ID was retained to ensure anonymous analysis.

### Statistical Analysis

Descriptive statistics were generated to present the demographic and medical characteristics of participants. The difference of the average mean between each level of patient satisfaction was analyzed using an ANOVA performed on SPSS 26 (IBM Corp) software. The caregiver acceptance questionnaire was analyzed by computing the proportion of each response for a given item. UTAUT scores were reported as the average score given to all items of a given dimension for all participants. System logs were analyzed by looking at the number of SMS text messages sent each month during the observation period.

## Results

### Demographics

Patient questionnaires were distributed between March 13 and April 28, 2017, by a total of 20 nurses during two different shifts (7:30 AM to 4 PM and 3 PM to 11:30 PM). The total number of collected questionnaires was 100. One patient was excluded because he visited the unit twice during the study period. The questionnaire took an average of 10 minutes to complete. Baseline patient demographics and data related to the medical encounter are shown in Table 1. Of the 100 respondents, 87 (87%) were classified with an emergency level of 3, and 12 (12%) were classified in level 4. No patients were classified as levels 1 or 2 as these acuity triage levels require immediate care. The nurses’ questionnaire was proposed to all nurses of the unit (n=25) but was only completed by 10 nurses.

**Table 1:** Demographics of participants and information on their medical encounter.

		Participants (N=100)
Age (years), mean (SD)		38 (14.75)
**Sex, n (%)**		
	Male	60 (60)
	Female	40 (40)
**Triage level, n (%)**		
	3	87 (87)
	4	12 (12)
	Missing	1 (1)
**Wait time (hours), n (%)**		
	<1	32 (32)
	1-2	45 (45)
	2-3	14 (14)
	3-4	8 (8)
	>4	1 (1)

### Patient Satisfaction Questionnaire

As presented in Table 2, of the total 100 respondents, 97% (n=97) were satisfied with the SMS text message system. Among these, approximately 75% (n=75) were totally satisfied with their waiting time and 56% (n=56) were satisfied. A total of 79 (79%) respondents waited outside of the ED but inside the hospital, as the facility offers the possibility to wait in pleasant places such as the cafeteria, adjacent green spaces, and the meditation room. The fact that patients waited close to the ED was confirmed by the fact that 86% (n=86) of patients returned to the ED on foot. Therefore, 92 (92%) patients had sufficient time to return to the ED once recalled. A total of 95 (95%) patients considered the SMS text message to be clear and 72 (72%) did not feel particularly stressed waiting outside the ED.

**Table 2:** Questionnaire results.

			Participants (N=100), n (%)
**Where did you spend your time while waiting?**			
	At home	2 (2)	
	Outside the hospital	13 (13)	
	Inside the hospital	80 (80)	
	Other	6 (6)	
**How do you rate your actual wait time compared to the wait time announced by the nurses?**			
	Longer	25 (25)	
	Shorter	49 (49)	
	Equal	25 (25)	
	Not informed	1 (1)	
**The SMS text message content was clearly understandable?**			
	Totally agree	72 (72)	
	Partly agree	23 (23)	
	Neither agree nor disagree	4 (4)	
	Partly disagree	1 (1)	
	Totally disagree	0 (0)	
**Did you experience a feeling of stress linked to your absence from the emergency waiting room?**			
	Totally agree	8 (8)	
	Partly agree	10 (10)	
	Neither agree nor disagree	11 (11)	
	Partly disagree	22 (22)	
	Totally disagree	50 (50)	
**Did you have enough time to return to the emergency room after receiving the recall message?**			
	Totally agree	59 (59)	
	Partly agree	33 (33)	
	Neither agree nor disagree	4 (4)	
	Partly disagree	2 (2)	
	Totally disagree	0 (0)	
**How did you return to the emergency room after receiving the recall message?**			
	On foot	86 (86)	
	Public transport	8 (8)	
	Private transport	2 (2)	
	Other	4 (4)	
**Are you satisfied with the SMS text message recall service?**			
	Totally agree	75 (75)	
	Partly agree	22 (22)	
	Neither agree nor disagree	3 (3)	
	Partly disagree	0 (0)	
	Totally disagree	0 (0)	
**Were you satisfied with your waiting time?**			
	Totally agree	28 (28)	
	Partly agree	28 (28)	
	Neither agree nor disagree	20 (20)	
	Partly disagree	12 (12)	
	Totally disagree	11 (11)	

By asking patients what would be the minimum duration of expected wait that would trigger an interest to be enrolled in the system in a future encounter (Table 3), we found that 45 of the 100 (45%) patients were interested in the system regardless of the waiting time. After 30 minutes of expected waiting time, 75 (75%) patients were interested in the system, and 87 (87%) patients were interested after 1 hour.

**Table 3:** Patients interested in using the SMS text message system after n minutes.

Number of minutes	Patients, n (%)
0	45 (45)
10	51 (51)
20	63 (63)
30	75 (75)
40	75 (75)
50	75 (75)
60	87 (87)
70	87 (87)
80	88 (88)
90	93 (93)
100	93 (93)
110	93 (93)
120	100 (100)

### Satisfaction and Waiting Time

To determine whether wait time duration influenced the level of patient satisfaction with wait time, we assessed if the differences in mean wait time across the five wait time satisfaction modalities (ie, totally disagree, partly disagree, neither agree nor disagree, partly agree, and totally agree) were statistically significant (descriptive statistics are presented in Table 4). As the homogeneity of variance using Levene was not statistically significant (*P*=.42), meaning that the variances were equal across groups, an ANOVA was performed. We found no significant differences between wait time means as a function of wait time satisfaction (*P*=.32; *F*_4_=1.193).

**Table 4:** Average wait duration according to user satisfaction with wait time.

Satisfaction with wait time	Wait time (min), mean (SD)	Participants (N=100), n (%)
Totally disagree	86.9091 (64.28)	11 (11)
Partly disagree	105.0833 (58.78)	12 (12)
Neither agree nor disagree	101.5000 (58.06)	20 (20)
Partly agree	98.3929 (47.88)	28 (28)
Totally agree	75.0357 (46.22)	28 (28)
Total	91.9495 (53.10)	99 (99)

### Caregiver Acceptance Questionnaire

The UTAUT questionnaire distributed to all nurses using the system was completed by 10 nurses (20% participation rate; Table 5). Nurses emphasized the good ergonomics of the system as they rated effort expectancy with an average score of 6.0. This was also confirmed by the facilitating condition dimension, including the resources and knowledge necessary to use the system, which were ranked above 5. Behavioral intention was high as most users intended to use the system frequently in the future on a daily basis. The expected gain on performance was less obvious for respondents. Although most users found the system useful (mean 4.5, SD 1.9), they did not find that the system increased their productivity (mean 3.2, SD 1.6) or speed at work (mean 3.0, SD 1.4). Hedonic motivation ranked below 4 as users did not find the system enjoyable or fun to use. Finally, social influence scored the lowest (mean 2.3, SD 1.9) as all users did not observe a positive influence on their peers or hierarchy toward the use of the system.

**Table 5:** Score distribution for each UTAUT dimension.

UTAUT^a^ dimension	Nurses’ scores, n (%)								Score, mean (SD)
	1	2	3	4	5	6	7		
Performance expectancy (n=33)	1 (3)	13 (39)	4 (12)	4 (12)	5 (15)	4 (12)	2 (6)	3.6 (1.8)	
Effort expectancy (n=44)	0 (0)	3 (7)	0 (0)	3 (7)	6 (14)	9 (20)	23 (52)	6.0 (1.4)	
Social influence (n=19)	12 (63)	0 (0)	0 (0)	4 (21)	2 (11)	0 (0)	1 (5)	2.3 (1.9)	
Facilitating condition (n=41)	1 (2)	3 (7)	1 (2)	6 (15)	11 (27)	6 (15)	13 (32)	5.3 (1.6)	
Hedonic motivation (n=24)	5 (21)	5 (21)	3 (13)	4 (17)	2 (8)	2 (8)	3 (13)	3.5 (2.1)	
Behavioral intention (n=30)	0 (0)	2 (7)	3 (10)	4 (13)	6 (20)	6 (20)	9 (30)	5.3 (1.6)	

^a^UTAUT: Unified Theory of Acceptance and Use of Technology.

### Log Analysis

**Figure 2:**
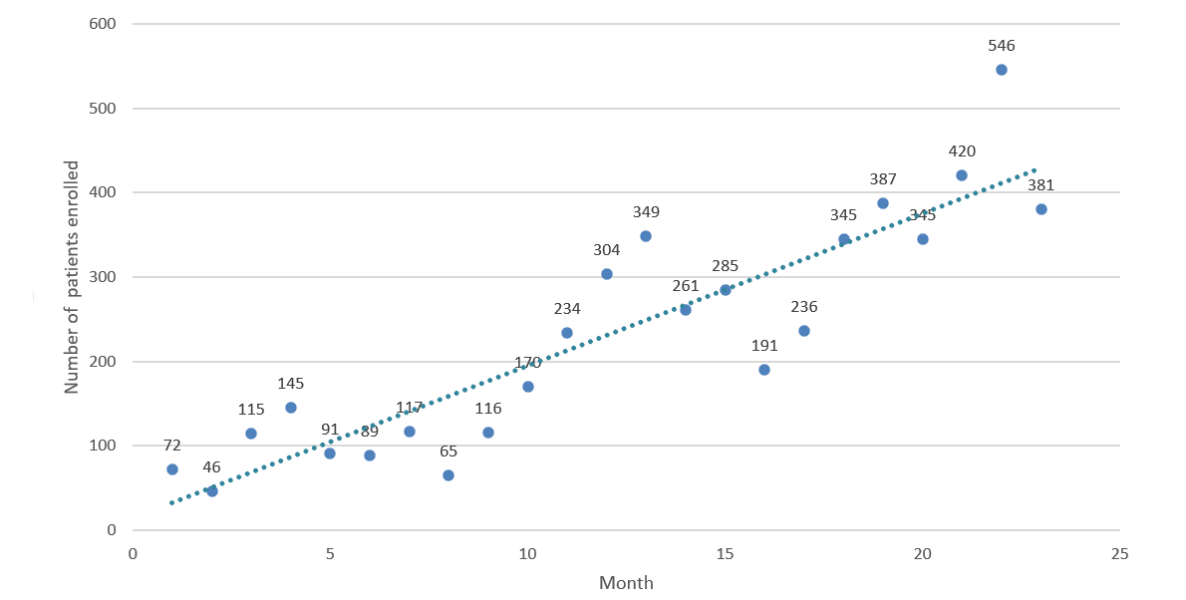
Number of unique patients enrolled in the SMS text message system each month (October 1, 2017, to August 31, 2019) and linear trend.

Figure 2 shows the number of unique patients entered into the SMS text message system from its introduction on October 1, 2017, to August 31, 2019. Although not always continuous, there was a clear trend of an increase in system use over time, ranging from 46 patients enrolled in November 2017 to 546 in July 2019. This corresponds to the trend of a linear function (18*x + 14) meaning that each month 18 additional patients are included in the system.

## Discussion

### Principal Findings

In this study, we found that 87 of the 100 (87%) patients with low-to-moderate urgency were interested in waiting outside the ED waiting room when the expected wait time was 1 hour or more. In a previous study, we observed that patients perceived the wait to be acceptable if it did not exceed 1 hour [21]. After 2 hours, they preferred to leave the ED before seeing a physician [22]. We observed that waiting outside the emergency room was perceived as a source of stress for <20% of participants, possibly related to the perceived reduced control over the situation when outside the room. Indeed, patients waiting outside the waiting room have no view on the current situation and can easily imagine being forgotten by ED staff [23]. Patients may also be concerned that their condition may worsen [24]. Thus, it may be worthwhile to send a recall SMS text message at regular intervals to indicate the patient’s current place in the waiting queue to provide reassurance about their position and the progression of the ED process [25]. The messages could also inform the patient about the proper actions in case their condition worsens, such as approaching a specific person or to go to a specific desk to advise staff. This type of concern has already been highlighted in another report showing that some patients want to remain visible to the caregiver to avoid being forgotten [26].

By comparing the relationship between wait time and satisfaction with our previous study [21] performed in a similar setting, we observed a reduced negative influence of the average wait time on patient satisfaction. Whereas in previous studies longer wait times led to significantly less satisfied patients, this relationship was no longer observed in our study [27]. This may indicate that patients are less concerned about the length of wait if they can wait in another location than in a waiting room where they have little to do but remain seated until they are taken care of. This correlates well with our results indicating that most patients were willing to use the system if the wait was longer than 1 hour.

Use of the system by the nursing staff began at a low frequency but increased steadily over time. Nurses’ initial reaction to the system was negative or neutral, and they initially perceived the tool as an additional burden to their workload. This phenomenon has already been observed in other studies [28,29]. The use of the system by many patients allowed it to predict potential benefits of the tool, such as reduced interruptions due to inpatient patients and reduced aggressive behavior in the waiting room due to long wait times [30]. However, informal feedback from nurses using the system highlighted the difficulty of using it when the ED was crowded. This is probably because busy nurses have less time to use the system in addition to regular duties that results in a contradictory effect that prevents the system from being used when it would be most useful. There are two solutions that can be considered to deal with this problem. Either the system can be used by administrative staff or the system can be automated. At our institution, the drive to develop this system has been a top-down process, and we plan to employ administrative workers to offload these tasks from nursing staff.

### Limitations

A strong limitation of the paper is the absence of a strict control group. To compare the effect of our intervention on the relationship between wait time and patient satisfaction, we used the results of a previous study [21] conducted in the same setting where we explored the factors associated with wait perception as a preintervention finding. However, since the questionnaire was not the same, the comparison is limited. The selection of patients based on their interest in using the SMS text message system must be taken into account as it certainly has an impact on the high satisfaction rate, as well as on the low-stress rate related to a wait outside the ED. Indeed, a patient with a high-stress level could refuse to use the system.

Another limitation is the use of a questionnaire of our own design. Since the questionnaire has not been scientifically validated, we cannot guarantee that it measured accurately the investigated constructs. Unfortunately, we did not record the acceptance rate of the system, and it would have been interesting to see how many patients refused the system and preferred to stay in the waiting room. The low participation rate of nurses is also a limitation, and it will be useful to conduct a further survey following the training of administrative staff to take over tasks.

### Conclusions

Waiting in the emergency waiting room is a source of frustration for the patient. In addition to the increase in aggressive attitudes in some patients when the ED waiting room is crowded, it also puts patients at risk of hospital-acquired infections. We observed a high level of satisfaction with our SMS text message recall system, allowing a wait outside the ED, but the adoption was more difficult among nurses. Relying on further automation of the system may be an interesting solution to reduce caregiver workload, but this must be done with caution given the high unpredictability of the ED waiting process.

## Abbreviations

ED: emergency department
UTAUT: Unified Theory of Acceptance and Use of Technology
